# Association of Long-term Use of Antihypertensive Medications With Late Outcomes Among Patients With Aortic Dissection

**DOI:** 10.1001/jamanetworkopen.2021.0469

**Published:** 2021-03-03

**Authors:** Shao-Wei Chen, Yi-Hsin Chan, Chia-Pin Lin, Victor Chien-Chia Wu, Yu-Ting Cheng, Dong-Yi Chen, Shang-Hung Chang, Kuo-Chun Hung, Pao-Hsien Chu, An-Hsun Chou

**Affiliations:** 1 Division of Thoracic and Cardiovascular Surgery, Department of Surgery, Chang Gung Memorial Hospital, Linkou Medical Center, Chang Gung University, Taoyuan City, Taiwan; 2 Center for Big Data Analytics and Statistics, Chang Gung Memorial Hospital, Linkou Medical Center, Taoyuan City, Taiwan; 3 Department of Cardiology, Chang Gung Memorial Hospital, Linkou Medical Center, Taoyuan City, Taiwan; 4 Department of Anesthesiology, Chang Gung Memorial Hospital, Linkou Medical Center, Chang Gung University, Taoyuan City, Taiwan

## Abstract

**Importance:**

The associations between long-term treatment of aortic dissection with various medications and late patient outcomes are poorly understood.

**Objective:**

To compare late outcomes after long-term use of β-blockers, angiotensin-converting enzyme inhibitors (ACEIs), angiotensin receptor blockers (ARBs), or other antihypertensive medications (controls) among patients treated for aortic dissection.

**Design, Setting, and Participants:**

This population-based retrospective cohort study using the National Health Insurance Research Database in Taiwan included 6978 adult patients with a first-ever aortic dissection who survived to hospital discharge during the period between January 1, 2001, and December 31, 2013, and who received during the first 90 days after discharge a prescription for an ACEI, ARB, β-blocker, or at least 1 other antihypertensive medication. Data analysis was conducted from July 2019 to June 2020.

**Exposure:**

Long-term use of β-blockers, ACEIs, or ARBs, with use of other antihypertensive medications as a control.

**Main Outcomes and Measures:**

The primary outcomes of interest were all-cause mortality, death due to aortic aneurism or dissection, later aortic operation, major adverse cardiac and cerebrovascular events, hospital readmission, and new-onset dialysis.

**Results:**

Of 6978 total participants, 3492 received a β-blocker, 1729 received an ACEI or ARB, and 1757 received another antihypertension drug. Compared with patients in the other 2 groups, those in the β-blocker group were younger (mean [SD] age, 62.1 [13.9] years vs 68.7 [13.5] years for ACEIs or ARBs and 69.9 [13.8] years for controls) and comprised more male patients (2520 [72.2%] vs 1161 [67.1%] for ACEIs or ARBs and 1224 [69.7%] for controls). The prevalence of medicated hypertension was highest in the ACEI or ARB group (1039 patients [60.1%]), followed by the control group (896 patients [51.0%]), and was lowest in the β-blocker group (1577 patients [45.2%]). Patients who underwent surgery for type A aortic dissection were more likely to be prescribed β-blockers (1134 patients [32.5%]) than an ACEI or ARB (309 patients [17.9%]) or another antihypertension medication (376 patients [21.4%]). After adjusting for multiple propensity scores, there were no significant differences in any of the clinical characteristics among the 3 groups. No differences in the risks for all outcomes were observed between the ACEI or ARB and β-blocker groups. The risk of all-cause hospital readmission was significantly lower in the ACEI or ARB group (subdistribution hazard ratio [HR], 0.92; 95% CI, 0.84-0.997) and β-blocker group (subdistribution HR, 0.87; 95% CI, 0.81-0.94) than in the control group. Moreover, the risk of all-cause mortality was lower in the ACEI or ARB group (HR, 0.79; 95% CI, 0.71-0.89) and the β-blocker group (HR, 0.82; 95% CI, 0.73-0.91) than in the control group. In addition, the risk of all-cause mortality was lower in the ARB group than in the ACEI group (HR, 0.85; 95% CI, 0.76-0.95).

**Conclusions and Relevance:**

The use of β-blockers, ACEIs, or ARBs was associated with benefits in the long-term treatment of aortic dissection.

## Key Points

**Question:**

Is there an association between long-term medication therapy and late outcomes among patients with aortic dissection?

**Findings:**

In this population-based cohort study of 6978 adults with aortic dissection, the risk of all-cause mortality was lower among patients prescribed an angiotensin-converting enzyme inhibitor (ACEI) or an angiotensin receptor blocker (ARB) and those prescribed a β-blocker than among those prescribed other antihypertension agents. The risk of all-cause mortality was lower in the ARB group than in the ACEI group in long-term follow-up.

**Meaning:**

Compared with other antihypertension agents, β-blockers and ACEIs or ARBs may be associated with benefits in the long-term treatment of aortic dissection.

## Teaser

This cohort study using the National Health Insurance Research Database in Taiwan compares outcomes after long-term use of β-blockers, angiotensin-converting enzyme inhibitors, angiotensin receptor blockers, or other antihypertensive medications among adults treated for aortic dissection.

## Introduction

In aortic dissection (AD), long-term medical therapy is usually prescribed to decrease the stress on the aortic wall and prevent aortic expansion or rupture.^1^ Medication therapy for AD is still based on historical observational studies and expert opinion. Guidelines from the European Society of Cardiology,^2^ American College of Cardiology/American Heart Association,^3^ and Japanese Circulation Society^4^ recommend β-blockers for the initial management of acute AD. Observational studies show that the use of β-blockers may decrease the aortic dilatation rate in aortic disease.^5,6^

Emerging evidence has linked the renin-angiotensin system to the development of aortic aneurysms (AAs). In genetic studies, polymorphisms of the angiotensin-converting enzyme (ACE) have been associated with AA.^7^ For patients with Marfan syndrome, treatment with an ACE inhibitor (ACEI) or an angiotensin receptor blocker (ARB) appears to decrease the progress of aortic dilatation and its complications.^8,9^ Several animal studies have shown that treatment with an ACEI or ARB slows AA progression and prevents rupture.^10,11^ A randomized clinical trial assessing the use of irbesartan for Marfan syndrome showed that ARBs decreased aortic expansion.^12^ However, no randomized clinical trial has compared the effects of long-term treatment with β-blockers, ACEIs, or ARBs with those of other antihypertensive medications after AD. Therefore, the present nationwide retrospective cohort study was conducted to compare the long-term use of β-blockers, ACEIs, or ARBs with that of other antihypertensive medications and their association with late outcomes among patients with AD.

## Methods

### Data Source

We designed a population-based retrospective cohort study by extracting data from the National Health Insurance Research Database, maintained by the Taiwan National Health Research Institute. Taiwan launched a National Health Insurance (NHI) program on March 1, 1995. The NHI system offers follow-up information on medications as well as on admission, outpatient clinic, and emergency department visit records of the Taiwanese population. This study followed the Strengthening the Reporting of Observational Studies in Epidemiology (STROBE) reporting guideline for cohort studies. The study was approved by the Chang Gung Memorial Hospital ethics board, which waived the requirement for obtaining informed consent because this was a retrospective study. No one received compensation or was offered any incentive for participating in this study.

In Taiwan, after receiving treatment for life-threatening diseases, patients receive discharge medications and are advised to attend at least 1 follow-up visit at the outpatient clinic to receive their prescriptions within 1 month after discharge and then visit within every 3 months afterward. Accurate health reimbursement records, ensured by prescriptions of medications, are followed up with appropriate examinations and indications. False reports of a diagnosis and inadequate indications for a prescription incur a severe penalty from the Bureau of NHI.

### Study Population

**Figure 1.**
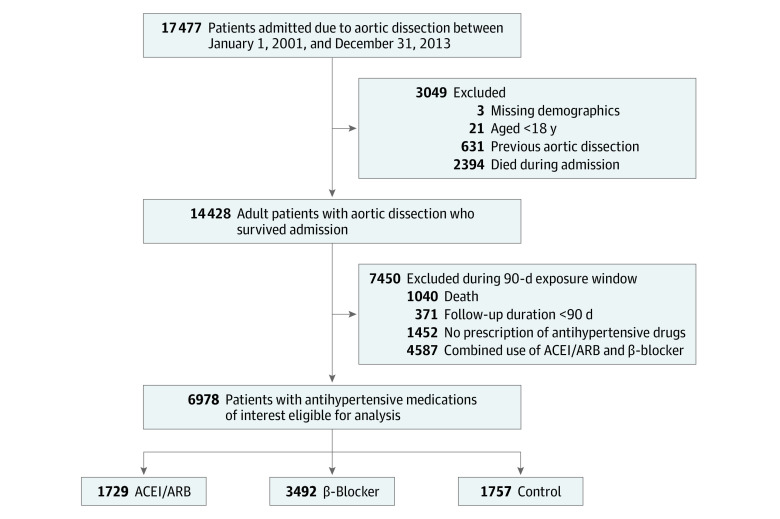
Patient Selection Flowchart ACEI indicates angiotensin-converting enzyme inhibitor; ARB, angiotensin receptor blocker.

The *International Classification of Diseases, Ninth Revision, Clinical Modification* diagnostic code 441.0x was used to identify patients who were diagnosed as having AD. Figure 1 is a flowchart describing patient selection. In total, 6978 patients with a first-ever AD were eligible for analysis between January 1, 2001, and December 31, 2013. Patients were placed into 1 of 3 groups based on the prescription records of the claims data from both outpatient visits and the refills in the pharmacy for chronic illness during the first 90 days after discharge: (1) ACEI or ARB, (2) β-blocker, or (3) the control group comprising patients who received at least 1 other antihypertensive drug. Medication use was ascertained by requiring each patient to have at least 2 prescriptions written during outpatient visits (a maximum of 30 days for each prescription) or 1 refill prescription for chronic illness filled at a pharmacy (a maximum of 60 days for each prescription).

### Outcomes

The first primary outcome was all-cause mortality, which was defined as withdrawal from the NHI program.^13^ Death due to AD or AA was detected by examining the cause of death using diagnoses in the inpatient records or emergency department visits within 7 days before the date of death.^13^ Major adverse cardiac and cerebrovascular events (MACCEs) included acute myocardial infarction, stroke, and cardiovascular death. The occurrence of stroke and acute myocardial infarction was defined as a principal discharge diagnosis. Cardiovascular death was defined based on the criterion of the standardized definitions for cardiovascular and stroke end point events in clinical trials by the US Food and Drug Administration in the United States.^14^ Permanent dialysis was identified by possessing a catastrophic illness certificate verified by the Bureau of NHI. All patients were followed up until December 31, 2013; the date of event occurrence; or date of death or whichever came first. When an individual had multiple events at different times (eg, a stroke and then an acute myocardial infarction), they were not censored at the first event (stroke) when analyzing the later event (acute myocardial infarction).

### Covariates

The covariates were age, sex, 13 selected comorbidities, the Charlson Comorbidity Index score, hospital level of index admission, aortic surgery at the index admission, extension of aortic surgery, additional cardiac surgical procedures, 7 postoperative antihypertensive agents other than an ACEIs, ARBs, and β-blockers, and 5 types of other medications. Comorbidities were defined as at least 2 outpatient diagnoses or 1 inpatient diagnosis in the previous year. The details associated with the *International Classification of Diseases, Ninth Revision, Clinical Modification* diagnostic codes are given in eTable 1 in the Supplement.

### Statistical Analysis

The baseline characteristics of the patients were compared using 1-way analysis of variance for continuous variables (except for the Charlson Comorbidity Index score) and the χ^2^ test for categorical variables. The Charlson Comorbidity Index score was compared among groups using the nonparametric Kruskal-Wallis test because of the skewed distribution. Instead of a traditional multivariable adjustment, an adjustment using multiple propensity scores was adopted. First, a multivariable multinomial logistic model was established by treating the study groups (3 categories) as outcome variables and all baseline characteristics (not including the clinical outcomes of interest) as covariates with forced entry (eTable 2 in the Supplement), with the follow-up year replacing the index date. As a result, 3 estimated probabilities (and propensity scores) for each individual with regard to membership in a given group were generated. The index date was also included in the calculation of propensity scores to enable the follow-up duration to be potentially equal. Group differences associated with the baseline characteristics could be minimized when any 2 of the 3 propensity scores were adjusted.^15^

To evaluate the balance of the baseline characteristics among the study groups after adjustment for multiple propensity scores, a series of multinomial logistic models were applied by treating the study groups as the outcome variables and each of the baseline characteristics as a covariate (termed *multivariate analysis*). An observation of nonsignificance (*P* > .05) suggested that there was no significant difference among the study groups after propensity score adjustment.

The risks of all-cause mortality among the study groups were compared with a Cox proportional hazards regression model. The survival analyses were additionally adjusted for multiple propensity scores. To detect the possibility of residual confounding, we used 2 negative control outcomes: fracture and malignant neoplasm.^16^ As a secondary analysis, we compared the outcomes between the ARB and ACEI groups. We performed inverse probability of treatment weighting with a stabilized weight based on the propensity score to estimate the mean treatment. The study group (ARB vs ACEI) was the only explanatory variable in the survival analyses.

In addition to the head-to-head comparison design (the primary analysis), we also conducted a sensitivity analysis by treating medication use as a time-varying exposure. The status of drug use was reassessed per 3 months during follow-up after the index date. Owing to the potential for treatment indication bias, comparisons were made only for the exposure periods with antihypertensive treatments. The first analysis compared ACEIs or ARBs alone, β-blockers alone, and the combination of both ACEIs or ARBs and β-blockers. All baseline characteristics, other antihypertensive agents (including calcium channel blockers [CCBs], α-blockers, thiazide, loop diuretics, spironolactone, vasodilators, and nitrates), and other medications were also treated as time-varying covariates and adjusted in the model. The second analysis compared ARBs alone with ACEIs alone, and the other antihypertensive agents (including β-blockers, CCBs, α-blockers, thiazide, loop diuretics, spironolactone, vasodilators, and nitrates) were adjusted in the model. In addition, we compared ACEIs or ARBs alone, β-blockers alone, and CCBs alone in another head-to-head comparison design. The other antihypertensive agents (including α-blockers, thiazide, loop diuretics, spironolactone, vasodilators, and nitrates) were adjusted in the model. Only 2 primary outcomes (all-cause mortality and death due to AD or AA) were analyzed in the aforementioned sensitivity analysis and additional analyses.

A 2-sided *P* < .05 was considered statistically significant, and no adjustments for multiple testing (multiplicity) were made. All statistical analyses were performed from July 2019 to June 2020 using SAS, version 9.4 (SAS Institute Inc).The direct-adjusted (estimated) survival was derived from the multivariable Cox proportional hazards regression model with the SAS macro ADJSURV.^17^ The direct-adjusted (estimated) cumulative incidence function was obtained using the Fine-Gray model with the macro CIFCOX.^18^

## Results

### Study Population Characteristics

The clinical characteristics of patients with AD stratified by their use of ACEIs or ARBs, β-blockers, or other antihypertensive agents are given in eTable 2 in the Supplement. In total, 1729 patients were prescribed ACEIs or ARBs, 3492 patients were prescribed β-blockers, and 1757 patients were prescribed a different antihypertension agent. In the univariate analysis, there were significant differences in most of the clinical characteristics among these 3 study groups. Patients in the β-blocker group were substantially younger (mean [SD] age, 62.1 [13.9] years for β-blockers, 68.7 [13.5] years for ACEIs or ARBs, and 69.9 [13.8] years for other antihypertensive agents) and composed predominantly of male patients (2520 patients [72.2%] for β-blockers, 1161 patients [67.1%] for ACEIs or ARBs, and 1224 patients [69.7%] for other antihypertensive agents). The prevalence of medicated hypertension was highest in the ACEI or ARB group (1039 patients [60.1%]), followed by the control group (896 patients [51.0%]), and was lowest in the β-blocker group (1577 patients [45.2%]). Patients who underwent surgery for type A AD were more likely to be prescribed β-blockers (1134 patients [32.5%]) than other antihypertensive agents (376 patients [21.4%]) and ACEIs or ARBs (309 patients [17.9%]). After adjustment for multiple propensity scores, there were no significant differences in any of the clinical characteristics among the 3 groups.

### Antihypertensive Drugs Prescribed for AD Across the Study Years

The use of β-blockers stably increased from 2001 to 2013 (52%-64.2%; *P* < .001 for trend). The use of an ACEI or ARB as a combined group also increased from 2001 to 2013 (39.6%-51.2%; *P* < .001 for trend). The use of an ARB assessed alone steadily increased from 2001 to 2013 (18.8%-47.2%; *P* < .001 for trend), whereas the use of an ACEI assessed alone decreased across these years (22.4%-5.0%; *P* < .001 for trend) (eFigure 1 and eTable 3 in the Supplement). The trends in the use of other antihypertensive agents across those same years are provided in eFigure 2 and eTable 3 in the Supplement.

### Late Outcomes of Interest

**Figure 2.**
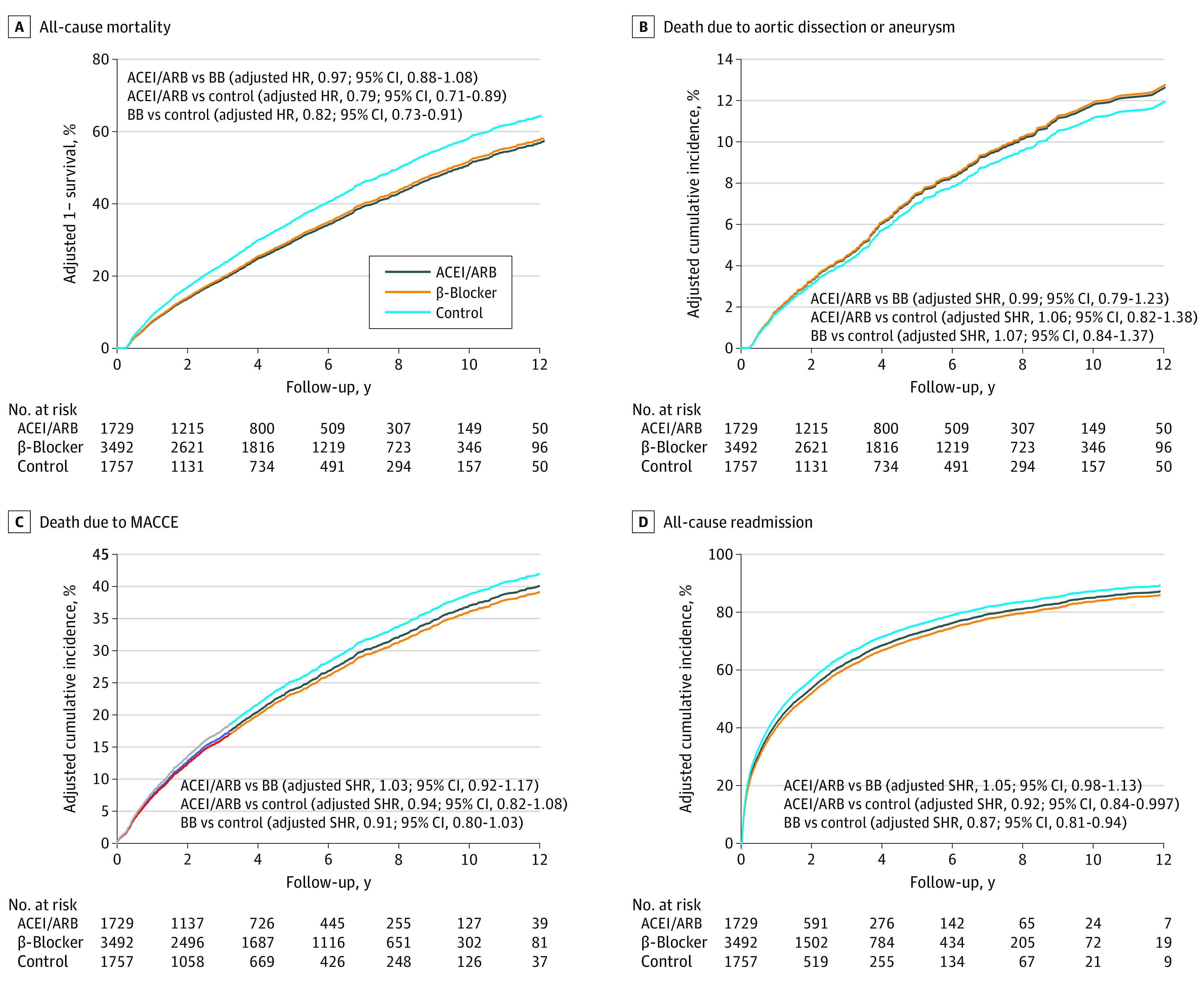
Direct-Adjusted (Estimated) Survival of All-Cause Mortality (A) and Direct-Adjusted (Estimated) Cumulative Incidence Function of Death Due to Aortic Dissection or Aneurysm (B), Major Cardiac and Cerebrovascular Events (MACCEs) (C), and All-Cause Readmission Among Patients With Other Antihypertensive Agents (Control) (D) ACEI indicates angiotensin-converting enzyme inhibitor; ARB, angiotensin receptor blocker; BB, β-blocker; HR, hazard ratio; and SHR, subdistribution hazard ratio.

The outcomes of interest, including all-cause mortality, death due to AD or AA, repeated aortic surgery, MACCE, hospital readmission due to any cause, and new-onset dialysis, were not significantly different between the ACEI or ARB group and the β-blocker group (Table 1). However, the risk of all-cause mortality was lower in the ACEI or ARB group (hazard ratio [HR], 0.79; 95% CI, 0.71-0.89) and the β-blocker group (HR, 0.82; 95% CI, 0.73-0.91) than in the control group (Table 1; Figure 2A). Although death due to AD or AA and risk of composite outcomes (MACCE) were not significantly different among the 3 groups, the risk of all-cause hospital readmission was significantly lower in the ACEI or ARB group (subdistribution HR, 0.92; 95% CI, 0.84-0.997) and the β-blocker group (subdistribution HR, 0.87; 95% CI, 0.81-0.94) than in the control group (Table 1; Figure 2B-D). No difference in the risks of negative control outcomes (ie, fracture or malignant neoplasm) was observed among groups (Table 1). Subgroup analyses of all-cause mortality and of death due to AD or AA by type A or type B dissection were also performed, and the results are shown in eTable 4 and eTable 5 in the Supplement.

**Table 1.** Time-to-Event Outcome Analysis During Follow-up Stratified by Antihypertensive Drug

Outcome	Event, No. (%) of patients			Propensity score–adjusted HR or SHR (95% CI)		
	ACEI or ARB (n = 1729)	β-Blocker (n = 3492)	Control (n = 1757)	ACEI or ARB vs β-blocker	ACEI or ARB vs control	β-Blocker vs control
All-cause mortality	642 (37.1)	985 (28.2)	825 (47.0)	0.97 (0.88-1.08)	0.79 (0.71-0.89)^a^	0.82 (0.73-0.91) ^a^
Death due to aortic aneurysm or dissection	140 (8.1)	241 (6.9)	151 (8.6)	0.99 (0.79-1.23)	1.06 (0.82-1.38)	1.07 (0.84-1.37)
Repeat aortic surgery	119 (6.9)	345 (9.9)	125 (7.1)	0.90 (0.72-1.11)	0.95 (0.73-1.23)	1.06 (0.85-1.32)
MACCE	477 (27.6)	760 (21.8)	541 (30.8)	1.03 (0.92-1.17)	0.94 (0.82-1.08)	0.91 (0.80-1.03)
Acute myocardial infarction	37 (2.1)	58 (1.7)	44 (2.5)	0.90 (0.57-1.42)	0.86 (0.54-1.37)	0.96 (0.61-1.52)
Stroke	182 (10.5)	323 (9.2)	198 (11.3)	1.01 (0.83-1.23)	1.01 (0.81-1.26)	1.00 (0.82-1.22)
Cardiovascular death	341 (19.7)	503 (14.4)	388 (22.1)	1.05 (0.91-1.22)	0.96 (0.81-1.14)	0.92 (0.78-1.07)
Readmission due to any cause	1269 (73.4)	2303 (66.0)	1354 (77.1)	1.05 (0.98-1.13)	0.92 (0.84-0.997)^a^	0.87 (0.81-0.94)^a^
New-onset dialysis	51 (2.9)	132 (3.8)	56 (3.2)	0.82 (0.59-1.14)	0.99 (0.66-1.49)	1.20 (0.83-1.75)
Negative control outcome						
Fracture	192 (11.1)	341 (9.8)	207 (11.8)	0.95 (0.78-1.14)	0.95 (0.76-1.18)	1.00 (0.82-1.22)
Malignant neoplasm	144 (8.3)	306 (8.8)	174 (9.9)	0.84 (0.69-1.04)	0.91 (0.71-1.16)	1.08 (0.87-1.34)

Abbreviations: ACEI, angiotensin-converting enzyme inhibitor; ARB, angiotensin receptor blocker; HR, hazard ratio; MACCE, major adverse cardiac and cerebrovascular event; SHR, subdistribution hazard ratio.

^a^ *P* < .05.

### Subgroup Analysis Comparing ARBs With ACEIs

**Figure 3.**
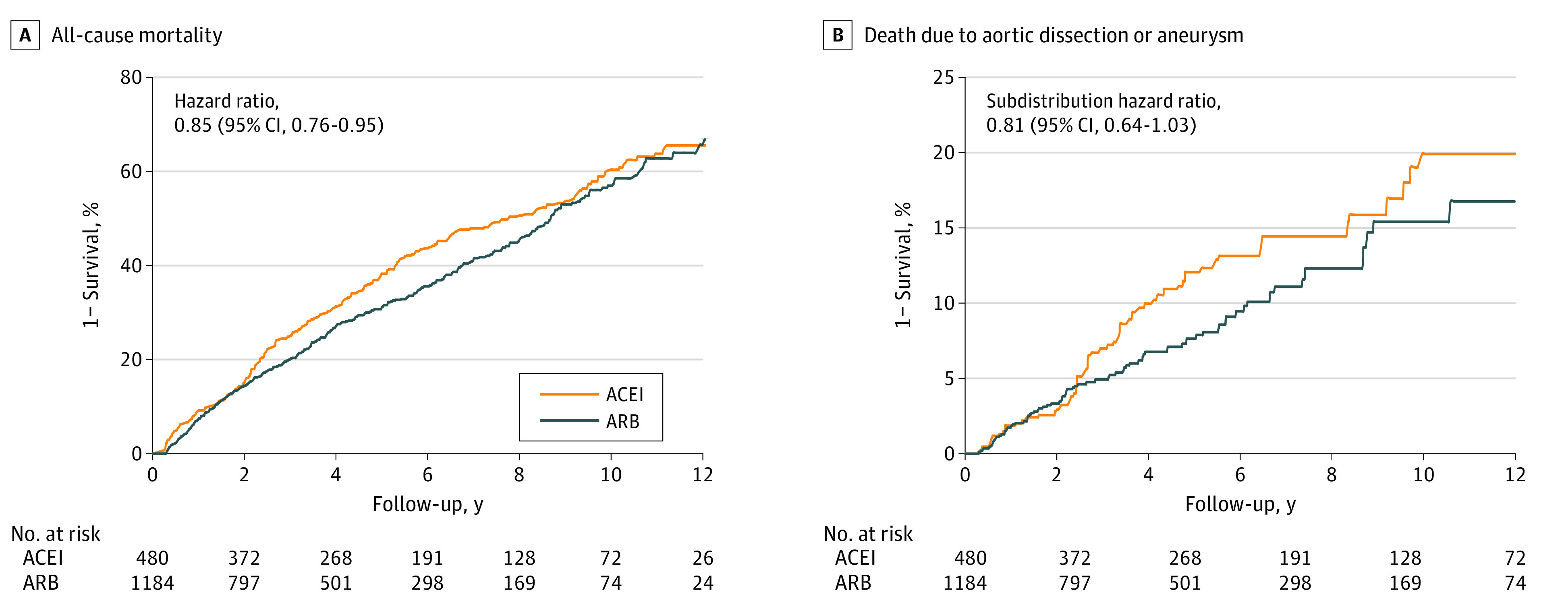
Kaplan-Meier Survival Curve of All-Cause Mortality (A) and Unadjusted Cumulative Incidence Function of Aortic Death (B) Among Patients Receiving an Angiotensin Receptor Blocker (ARB) vs Angiotensin-Converting Enzyme Inhibitor (ACEI) in the Inverse Probability of Treatment Weighting–Adjusted Cohort

The baseline characteristics of patients with AD by use of ARBs or ACEIs are given in eTable 6 in the Supplement. After inverse probability of treatment weighting, there were no substantial differences between the 2 groups. Table 2 shows the results of the outcome analysis. The risk of all-cause mortality was lower in the ARB group than in the ACEI group (HR, 0.85; 95% CI, 0.76-0.95) (Figure 3A). Death due to AD or AA appeared to be lower in the ARB group (subdistribution HR, 0.81; 95% CI, 0.64-1.03), although this finding was not statistically significant (*P* = .09) (Figure 3B). In addition, no difference in the risks of negative control outcomes was observed between groups (Table 2).

**Table 2.** Time-to-Event Outcome Analysis During Follow-up Stratified by the Use of ARBs or ACEIs

Outcome	Data before IPTW, No. (%) of patients		Data after IPTW			
	ARB (n = 1184)	ACEI (n = 480)	% of Patients		HR or SHR of ARB (95% CI)	*P* value
			ARB	ACEI		
All-cause mortality	377 (31.8)	245 (51.0)	36.3	39.8	0.85 (0.76-0.95)	.004
Death due to aortic aneurysm or dissection	80 (6.8)	55 (11.5)	7.6	9.1	0.81 (0.64-1.03)	.09
Repeat aortic surgery	83 (7.0)	34 (7.1)	7.2	5.7	1.24 (0.95-1.62)	.11
MACCE	288 (24.3)	171 (35.6)	26.8	26.8	1.00 (0.89-1.13)	.98
Acute myocardial infarction	22 (1.9)	15 (3.1)	2.2	2.1	1.01 (0.64-1.60)	.97
Stroke	111 (9.4)	62 (12.9)	9.9	9.5	1.02 (0.82-1.27)	.84
Cardiovascular death	205 (17.3)	124 (25.8)	19.6	19.2	1.01 (0.86-1.17)	.93
Readmission due to any cause	837 (70.7)	386 (80.4)	74.0	72.8	0.99 (0.91-1.07)	.70
New-onset dialysis	36 (3.0)	11 (2.3)	3.1	2.2	1.38 (0.90-2.11)	.14
Negative control outcome						
Fracture	119 (10.1)	61 (12.7)	11.1	11.1	0.99 (0.81-1.21)	.91
Malignant neoplasm	96 (8.1)	44 (9.2)	8.6	8.6	1.00 (0.79-1.26)	.99

Abbreviations: ACEI, angiotensin-converting enzyme inhibitor; ARB, angiotensin receptor blocker; HR, hazard ratio; IPTW, inverse probability of treatment weighting; MACCE, major adverse cardiac and cerebrovascular event; SHR, subdistribution hazard ratio.

### Sensitivity Analysis and Additional Analyses

Sensitivity analysis data on all-cause mortality and death due to AD or AA were obtained by using medication use as a time-varying exposure to treatment (eTable 7 in the Supplement). The use of either an ACEI or ARB alone or a β-blocker alone was associated with a lower but not statistically significant risk of both all-cause mortality and death due to AD or AA. However, the use of an ACEI or ARB combined with a β-blocker was associated with a significantly lower risk of both all-cause mortality (HR, 0.68; 95% CI, 0.56-0.83) and death due to AD or AA (HR, 0.64; 95% CI, 0.47-0.88). The results comparing ARBs with ACEIs were consistent with the primary analysis that ARBs were significantly associated with a lower risk of both all-cause mortality (HR, 0.72; 95% CI, 0.63-0.82) and death due to AD or AA (HR, 0.71; 95% CI, 0.58-0.87) (eTable 8 in the Supplement).

A flowchart for inclusion of patients with use of an ACEI or ARB, a β-blocker, or a CCB is provided in eFigure 3 in the Supplement, and eTable 9 in the Supplement shows baseline data for patients in these 3 groups. After adjustment for the 2 propensity scores, the results indicated that, compared with the use of a CCB, the use of an ARB or ACEI (HR, 0.76; 95% CI, 0.65-0.88) or of a β-blocker (HR, 0.86; 95% CI, 0.75-0.995) was associated with a significantly lower risk of all-cause mortality. The use of an ARB or ACEI rather than a β-blocker was associated with a lower risk of death due to AD or AA (HR, 0.67; 95% CI, 0.48–0.94) (eTable 10 in the Supplement).

## Discussion

In the present study, we found that both β-blockers and ACEIs or ARBs were associated with a lower risk of all-cause mortality and with hospital readmission due to any cause compared with their propensity score–matched controls. The risk of all-cause mortality was lower in the ARB-treated group than in the ACEI-treated group.

Although observational studies have shown that the use of β-blockers may decrease the aortic dilatation rate in aortic disease, to our knowledge, no randomized clinical trial has compared the use of β-blockers with the use of other antihypertensive drugs for the long-term treatment of AD. Emerging evidence has suggested that angiotensin II levels in the renin-angiotensin system are markedly increased in human AA through the ACE-dependent and the chymase-dependent pathways.^19,20^ Limited experimental and clinical studies have indicated that ACEIs and ARBs inhibit growth of AAs.^21,22^ However, these findings are discordant with another study indicating that ACEIs may be assocaited with faster abdominal AA growth.^23^ In our study, similar to the β-blocker group, the ACEI or ARB group had lower risks than the control cohort of all-cause mortality and hospital readmission due to any cause.

We also found that ARBs were associated with lower risk than ACEIs of all-cause mortality. The insights gained from study of Marfan syndrome–related fibrillin 1 highlight the potential role of transforming growth factor β (TGF-β) signaling in AA.^10,11,24^ The use of TGF-β neutralizing antibodies in fibrillin 1–deficient mice prevented AA in Marfan syndrome.^25^ Mice treated with losartan, an angiotensin II type 1 receptor (AT1R) blocker that antagonizes TGF-β signaling, exhibited no further aortic dilatation, thus suggesting the therapeutic efficacy of losartan against aneurysms.^26^

In the renin-angiotensin system, ACEIs show dual AT1R and AT2R blockade effects, whereas ARBs have an AT1R blockade effect.^26^ Only losartan uniquely inhibits TGF-β–mediated activation of extracellular signal–regulated kinase (ERK) by allowing for continued signaling through AT2Rs.^26^ These results indicate that losartan may be superior to ACEIs in preventing aortic root dilation through TGF-β–mediated ERK activation. Although such studies have suggested that ARBs are a promising agent in ameliorating the course of Marfan syndrome, the significance of ACEI or ARB treatment of other aneurysms is unclear. Losartan not only blocks TGF-β signaling but also prevents angiotensin II signaling by blocking AT1Rs, which may be activated in some forms of aneurysm.^26^ It is a reasonable speculation that ARBs may have beneficial effects in the treatment of more common nonhereditary AAs.

The study by Suzuki et al,^5^ which assessed a population from the International Registry of Acute Aortic Dissection, indicated that the use of β-blockers is associated with improved survival after surgery for type A AD and that the use of CCBs is associated with improved survival for patients medically treated for type B AD. The use of ACEIs did not show an association with survival. However, this benefit of CCBs has not been shown in other studies, and CCBs are not recommended for use in patients with inherited thoracic aortic disease. In our study, the use of ACEIs, ARBs, or β-blockers was associated with lower all-cause mortality in both type A and type B AD. Our results also showed that the use of ACEIs or ARBs and β-blockers, rather than use of CCBs, was associated with significantly lower risk of all-cause mortality. These disparate results may be because the analysis by Suzuki et al^5^ focused on patients discharged alive with medications, and their follow-up data included the use of those medications. However, we included only patients who received prescriptions 90 days after discharge, and our study used 2 research design methods for statistical analyses: head-to-head comparisons using a cohort study design and sensitivity tests using a time-varying exposure design.

### Limitations

Our study has several limitations. This was a retrospective population-based cohort study, and thus specific details of imaging findings, such as aortic size, extension of AD, or morphologic results, were not available. However, image reports are verified via the National Health Insurance Bureau to ensure medical consistency and that bias is kept to a minimum. Another limitation is that blood pressure levels and dosages of drugs are not recorded in the National Health Insurance Research Database, which may be a major confounder in our evaluation of clinical outcomes. However, we assessed numerous additional antihypertensive drugs in an effort to mitigate bias associated with different blood pressure levels. Finally, why some patients were given specific medical treatments (eg, to alleviate certain adverse effects) was unknown, which may have led to misclassification of exposures. Despite these limitations, we believe that this study provides results beneficial for clinicians selecting drugs for long-term treatment of AD.

## Conclusions

Compared with the control group, the use of β-blockers and ACEIs or ARBs was associated with lower risks of mortality and hospital readmission due to any cause. These data provide evidence that ACEI and ARB therapies may be alternatives to β-blocker use for the long-term treatment of AD.

## Notes

Supplement. **eTable 1.** *ICD-9-CM* Diagnostic Codes **eTable 2.** Baseline Characteristics of the Patients With Aortic Dissection According to Antihypertensive Drug Use **eTable 3.** Prescription of Antihypertensive Drugs Across The Study Years **eTable 4.** Outcomes of Primary Interest During the Follow-up in Patients With Type A Dissection **eTable 5.** Outcomes of Primary Interest During the Follow-up in Patients With Type B Dissection **eTable 6.** Baseline Characteristics of the Patients With Aortic Dissection According to the Use of ARBs or ACEIs **eTable 7.** Sensitivity Analysis by Treating Medication Use as Time-Varying Exposure (on Treatment) on the Primary Outcomes **eTable 8.** Sensitivity Analysis by Treating Medication Use as Time-Varying Exposure (on Treatment) on the Primary Outcomes, ARBs vs ACEIs **eTable 9.** Baseline characteristics of the Patients With Aortic Dissection According to Antihypertensive Drug Use **eTable 10.** Outcomes of Primary Interest During the Follow-up Comparing ACEIs/ARBs, β-Blocker and CCBs Users **eFigure 1.** The Trend of Prescription for Antihypertensive Agents of Primary Interest Across 2001 to 2013 in Taiwan **eFigure 2.** The Trend of Prescription for Antihypertensive Agents Not of Primary Interest Across 2001 to 2013 in Taiwan **eFigure 3.** The Flowchart for Inclusion of Patients With Use of ACEIs/ARBs, β-Blockers or CCBs Arm
