# Supplementary material for: Association of Long-term Use of Antihypertensive Medications With Late Outcomes Among Patients With Aortic Dissection
Source: JAMA Netw Open. 2021 Mar 3;4(3):e210469. doi: 10.1001/jamanetworkopen.2021.0469 (PMC7930924; doi:10.1001/jamanetworkopen.2021.0469)
Supplement: Supplement. — eTable 1. ICD-9-CM Diagnostic Codes eTable 2. Baseline Characteristics of the Patients With Aortic Dissection According to Antihypertensive Drug Use eTable 3. Prescription of Antihypertensive Drugs Across The Study Years eTable 4. Outcomes of Primary Interest During the Follow-up in Patients With Type A Dissection eTable 5. Outcomes of Primary Interest During the Follow-up in Patients With Type B Dissection eTable 6. Baseline Characteristics of the Patients With Aortic Dissection According to the Use of ARBs or ACEIs eTable 7. Sensitivity Analysis by Treating Medication Use as Time-Varying Exposure (on Treatment) on the Primary Outcomes eTable 8. Sensitivity Analysis by Treating Medication Use as Time-Varying Exposure (on Treatment) on the Primary Outcomes, ARBs vs ACEIs eTable 9. Baseline characteristics of the Patients With Aortic Dissection According to Antihypertensive Drug Use eTable 10. Outcomes of Primary Interest During the Follow-up Comparing ACEIs/ARBs, β-Blocker and CCBs Users eFigure 1. The Trend of Prescription for Antihypertensive Agents of Primary Interest Across 2001 to 2013 in Taiwan eFigure 2. The Trend of Prescription for Antihypertensive Agents Not of Primary Interest Across 2001 to 2013 in Taiwan eFigure 3. The Flowchart for Inclusion of Patients With Use of ACEIs/ARBs, β-Blockers or CCBs Arm [file jamanetwopen-e210469-s001.pdf]

# Supplementary Online Content

Chen SW, Chan YH, Lin CP, et al. Association of long-term use of antihypertensive medications with late outcomes among patients with aortic dissection. *JAMA Netw Open*. 2021;4(3):e210469. doi:10.1001/jamanetworkopen.2021.0469

**eTable 1.** *ICD-9-CM* Diagnostic Codes
**eTable 2.** Baseline Characteristics of the Patients With Aortic Dissection According to Antihypertensive Drug Use
**eTable 3.** Prescription of Antihypertensive Drugs Across The Study Years
**eTable 4.** Outcomes of Primary Interest During the Follow-up in Patients With Type A Dissection
**eTable 5.** Outcomes of Primary Interest During the Follow-up in Patients With Type B Dissection
**eTable 6**. Baseline Characteristics of the Patients With Aortic Dissection According to the Use of ARBs or ACEIs
**eTable 7.** Sensitivity Analysis by Treating Medication Use as Time-Varying Exposure (on Treatment) on the Primary Outcomes
**eTable 8.** Sensitivity Analysis by Treating Medication Use as Time-Varying Exposure (on Treatment) on the Primary Outcomes, ARBs vs ACEIs
**eTable 9**. Baseline characteristics of the Patients With Aortic Dissection According to Antihypertensive Drug Use
**eTable 10.** Outcomes of Primary Interest During the Follow-up Comparing ACEIs/ARBs, β-Blockers and CCBs Users
**eFigure 1.** The Trend of Prescription for Antihypertensive Agents of Primary Interest Across 2001 to 2013 in Taiwan
**eFigure 2.** The Trend of Prescription for Antihypertensive Agents Not of Primary Interest Across 2001 to 2013 in Taiwan
**eFigure 3.** The Flowchart for Inclusion of Patients With Use of ACEIs/ARBs, β-Blockers or CCBs Arm

This supplementary material has been provided by the authors to give readers additional information about their work.

© 2021 Chen SW et al. *JAMA Network Open*.

**eTable 1.** *ICD-9-CM* Diagnostic Codes

| Variable | ICD-9 CM Code |
|---|---|
| Aortic dissection | 441.0x |
| Hypertension | 401.xx-405.xx |
| Diabetes mellitus | 250.xx |
| Congestive heart failure | 428.xx |
| Coronary artery disease | 410.xx-414.xx |
| Old myocardial infarction | 410.xx, 412.xx |
| Peripheral arterial disease | 440.0x, 440.2x, 440.3x, 440.8x, 440.9x, 443x, 444.0x, 444.22, 444.8x, 447.8x, 447.9x |
| Atrial fibrillation | 427.31 |
| Old stroke | 430.xx-437.xx |
| Chronic kidney disease | 580.xx-589.xx, 403.xx-404.xx, 016.0x, 095.4x, 236.9x, 250.4x, 274.1x, 442.1x, 447.3x, 440.1x, 572.4x, 642.1x, 646.2x, 753.1x, 283.11, 403.01, 404.02, 446.21 |
| Dialysis | 585.xx (Catastrophic illness card) |
| Chronic obstructive pulmonary disease | 491.xx, 492.xx, 496.xx |
| Asthma | 493.xx |
| High degree AV blocker | 426.0, 426.12 |
| Aortic death | 441.xx, 798.xx |
| Acute myocardial infarction | 410.xx |
| Cardiovascular death | 390.xx-459.xx |
| Dialysis | 585.xx |
| Fracture | 800.xx-829.xx |

© 2021 Chen SW et al. *JAMA Network Open*.

| | |
|---|---|
| Malignancy | 140.xx-208.xx (Catastrophic illness card) |

Abbreviations: ICD-9 CM, International Classification of Diseases, Ninth Revision, Clinical Modification.

© 2021 Chen SW et al. *JAMA Network Open*.

**eTable 2.** Baseline Characteristics of the Patients With Aortic Dissection According to Antihypertensive Drug Use

| Variable | ACEI/ARB (*n* = 1,729) | β-blocker (*n* = 3,492) | Control (*n* = 1,757) | *P* value of univariate | *P* value of multivariate |
|---|---|---|---|---|---|
| Age (years) | 68.7 ± 13.5 | 62.1 ± 13.9 | 69.9 ± 13.8 | <0.001 | 0.761 |
| Male gender | 1,161 (67.1) | 2,520 (72.2) | 1,224 (69.7) | 0.001 | 0.999 |
| Comorbid conditions | | | | | |
| Hypertension | | | | <0.001 | 0.992 |
| None | 252 (14.6) | 687 (19.7) | 390 (22.2) | | |
| Without medication control | 438 (25.3) | 1,228 (35.2) | 471 (26.8) | | |
| With medication control | 1,039 (60.1) | 1,577 (45.2) | 896 (51.0) | | |
| Diabetes mellitus | 329 (19.0) | 400 (11.5) | 294 (16.7) | <0.001 | 0.992 |
| Congestive heart failure | 205 (11.9) | 182 (5.2) | 186 (10.6) | <0.001 | 0.996 |
| Coronary artery disease | 645 (37.3) | 961 (27.5) | 608 (34.6) | <0.001 | 0.963 |
| Old myocardial infarction | 105 (6.1) | 115 (3.3) | 99 (5.6) | <0.001 | 0.985 |
| Peripheral arterial disease | 122 (7.1) | 174 (5.0) | 98 (5.6) | 0.009 | 0.998 |
| Atrial fibrillation | 107 (6.2) | 152 (4.4) | 136 (7.7) | <0.001 | 0.918 |
| Old stroke | 302 (17.5) | 368 (10.5) | 308 (17.5) | <0.001 | 0.951 |
| Chronic kidney disease | 235 (13.6) | 555 (15.9) | 304 (17.3) | 0.009 | 0.930 |
| Dialysis | 22 (1.3) | 53 (1.5) | 38 (2.2) | 0.091 | 0.975 |
| COPD | 333 (19.3) | 277 (7.9) | 338 (19.2) | <0.001 | 0.893 |
| Asthma | 184 (10.6) | 158 (4.5) | 161 (9.2) | <0.001 | 0.980 |
| High degree AV blocker | 11 (0.6) | 13 (0.4) | 14 (0.8) | 0.120 | 0.996 |
| Charlson's Comorbidity Index score | 2 [1, 4] | 2 [1, 3] | 2 [1, 4] | <0.001 | 0.545 |
| Hospital level | | | | <0.001 | 0.933 |

© 2021 Chen SW et al. *JAMA Network Open*.

| | | | | | |
|---|---|---|---|---|---|
| Medical center (teaching hospital) | 1,007 (58.2) | 2,319 (66.4) | 974 (55.4) | | |
| Regional / district hospital | 722 (41.8) | 1,173 (33.6) | 783 (44.6) | | |
| Aortic surgery at the index admission | | | | <0.001 | 0.940 |
| Non-surgery | 1,355 (78.4) | 2,221 (63.6) | 1,315 (74.8) | | |
| Type A | 309 (17.9) | 1,134 (32.5) | 376 (21.4) | | |
| Type B | 65 (3.8) | 137 (3.9) | 66 (3.8) | | |
| Extension of aortic surgery | | | | | |
| Ascending aorta replacement | 266 (15.4) | 920 (26.3) | 311 (17.7) | <0.001 | 0.854 |
| Aortic arch replacement | 74 (4.3) | 327 (9.4) | 90 (5.1) | <0.001 | 0.885 |
| Aortic root replacement | 10 (0.6) | 81 (2.3) | 25 (1.4) | <0.001 | 0.593 |
| Additional cardiac surgery | | | | | |
| CABG | 46 (2.7) | 170 (4.9) | 74 (4.2) | 0.001 | 0.886 |
| Valve replacement | 46 (2.7) | 162 (4.6) | 52 (3.0) | <0.001 | 0.962 |
| Valve repair | 138 (8.0) | 537 (15.4) | 179 (10.2) | <0.001 | 0.800 |
| Post OP anti-HTN medication | | | | | |
| CCB | 916 (53.0) | 2,154 (61.7) | 855 (48.7) | <0.001 | 0.963 |
| Alpha-blocker | 215 (12.4) | 392 (11.2) | 187 (10.6) | 0.230 | 0.981 |
| Thiazide | 122 (7.1) | 180 (5.2) | 74 (4.2) | 0.001 | 0.999 |
| Loop diuretics | 365 (21.1) | 812 (23.3) | 438 (24.9) | 0.027 | 0.900 |
| Potassium-sparing diuretics | 67 (3.9) | 80 (2.3) | 86 (4.9) | <0.001 | 0.811 |
| Vasodilator | 531 (30.7) | 819 (23.5) | 555 (31.6) | <0.001 | 0.706 |
| Nitrate | 394 (22.8) | 628 (18.0) | 359 (20.4) | <0.001 | 0.981 |
| Number of anti-HTN drugs (not including ACEI/ARB and β-blocker) | 1.5 ± 1.2 | 1.5 ± 1.1 | 1.6 ± 0.9 | <0.001 | 0.882 |

© 2021 Chen SW et al. *JAMA Network Open*.

| | | | | | |
|---|---|---|---|---|---|
| Post OP other medication | | | | | |
| Statin | 197 (11.4) | 268 (7.7) | 126 (7.2) | <0.001 | 0.988 |
| Antiplatelet | 567 (32.8) | 797 (22.8) | 408 (23.2) | <0.001 | 0.980 |
| Anticoagulant | 127 (7.3) | 372 (10.7) | 132 (7.5) | <0.001 | 0.960 |
| OHA | 205 (11.9) | 233 (6.7) | 152 (8.7) | <0.001 | 0.969 |
| Insulin | 26 (1.5) | 16 (0.5) | 17 (1.0) | <0.001 | 0.953 |
| Follow up year | 4.5 ± 3.3 | 4.9 ± 3.4 | 4.2 ± 3.4 | <0.001 | 0.420 |

Abbreviations: ACEI, angiotensin converting enzyme inhibitor; ARB, angiotensin receptor blocker; COPD, chronic obstructive pulmonary disease; AV, atrioventricular; CABG, coronary artery bypass graft; OP, operation; HTN, hypertension; CCB, calcium channel blocker; OHA, oral hypoglycemic agent;

Value are given as number (%), median [$25^{th}$, $75^{th}$ percentile] or mean ± standard deviation.

© 2021 Chen SW et al. *JAMA Network Open*.

**eTable 3.** Prescription of Antihypertensive Drugs Across The Study Years

| | Prevalence of use (%) | | | | | | | | | | | | | *P* for trend |
|---|---|---|---|---|---|---|---|---|---|---|---|---|---|---|
| Drug | 2001 | 2002 | 2003 | 2004 | 2005 | 2006 | 2007 | 2008 | 2009 | 2010 | 2011 | 2012 | 2013 | |
| ACEI/ ARB | 39.6 | 42.1 | 45.6 | 43.5 | 43.9 | 45.2 | 48.7 | 51.3 | 57.3 | 50.7 | 50.5 | 52.4 | 51.2 | <0.001 |
| ACEI | 22.4 | 21.2 | 20.7 | 18.8 | 17.2 | 18.6 | 14.9 | 12.3 | 11.5 | 7.6 | 7.6 | 6.2 | 5.0 | <0.001 |
| ARB | 18.8 | 23.2 | 27.1 | 26.9 | 28.9 | 29.1 | 36.3 | 40.9 | 47.9 | 44.5 | 44.8 | 48.0 | 47.2 | <0.001 |
| Beta blocker | 52.0 | 59.2 | 60.2 | 56.6 | 63.5 | 63.5 | 61.5 | 62.0 | 65.5 | 64.7 | 64.1 | 64.0 | 64.2 | <0.001 |
| CCB | 47.0 | 49.5 | 51.8 | 50.6 | 53.8 | 57.2 | 55.0 | 55.5 | 56.4 | 56.7 | 53.4 | 53.8 | 48.0 | 0.029 |
| Alpha blocker | 11.7 | 12.9 | 11.9 | 11.7 | 10.1 | 10.2 | 10.8 | 12.1 | 13.8 | 12.3 | 13.9 | 14.2 | 12.9 | 0.008 |
| Thiazide | 10.4 | 7.0 | 6.7 | 9.2 | 9.9 | 9.2 | 4.9 | 6.4 | 5.9 | 3.5 | 3.9 | 3.1 | 2.8 | <0.001 |
| Loop diuretics | 20.2 | 19.2 | 20.2 | 17.7 | 16.6 | 18.2 | 21.1 | 19.7 | 17.2 | 20.5 | 17.7 | 16.9 | 17.5 | 0.136 |
| Spironolactone | 1.1 | 1.2 | 1.4 | 1.6 | 1.4 | 1.4 | 3.7 | 3.2 | 2.6 | 2.6 | 5.2 | 3.7 | 4.1 | <0.001 |
| Vasodilator | 26.8 | 26.5 | 23.0 | 23.8 | 25.4 | 23.8 | 23.3 | 22.2 | 19.7 | 21.0 | 21.0 | 20.3 | 19.9 | <0.001 |
| Nitrate | 20.0 | 20.9 | 18.2 | 18.5 | 20.4 | 18.5 | 18.2 | 16.4 | 13.8 | 16.6 | 14.1 | 14.8 | 13.8 | <0.001 |
| Number of total anti-HTN drugs | 2.3 ± 1.6 | 2.4 ± 1.6 | 2.4 ± 1.6 | 2.4 ± 1.6 | 2.5 ± 1.6 | 2.5 ± 1.6 | 2.5 ± 1.6 | 2.5 ± 1.6 | 2.5 ± 1.5 | 2.5 ± 1.5 | 2.5 ± 1.5 | 2.4 ± 1.5 | 2.4 ± 1.5 | 0.103 |

Abbreviations: ACEI, angiotensin converting enzyme inhibitor; ARB, angiotensin receptor blocker; CCB, calcium channel blocker; HTN, hypertension;

Value are given as number (%) or mean ± standard deviation.

© 2021 Chen SW et al. *JAMA Network Open*.

**eTable 4.** Outcomes of Primary Interest During the Follow-up in Patients With Type A Dissection

| | Number of event (%) | | | Propensity scores adjusted HR (95% CI) | | |
|---|---|---|---|---|---|---|
| Outcome | ACEI/ARB (*n* = 309) | β-blocker (*n* = 1,134) | Control (*n* = 376) | ACEI/ARB vs. β-blocker | ACEI/ARB vs. Control | β-blocker vs. Control |
| All-cause mortality | 62 (20.1) | 188 (16.6) | 102 (27.1) | 0.99 (0.74–1.33) | 0.84 (0.60–1.17) | 0.85 (0.65–1.11) |
| Aortic death | 15 (4.9) | 51 (4.5) | 22 (5.9) | 0.98 (0.55–1.76) | 0.83 (0.42–1.63) | 0.84 (0.48–1.45) |

Abbreviations: ACEI, angiotensin converting enzyme inhibitor; ARB, angiotensin receptor blocker; HR, hazard ratio; CI, confidence interval;
* *P* value <0.05.

© 2021 Chen SW et al. *JAMA Network Open*.

**eTable 5.** Outcomes of Primary Interest During the Follow-up in Patients With Type B Dissection

| | Number of event (%) | | | Propensity scores adjusted HR (95% CI) | | |
|---|---|---|---|---|---|---|
| Outcome | ACEI/ARB (*n* = 1,420) | β-blocker (*n* = 2,358) | Control (*n* = 1,381) | ACEI/ARB vs. β-blocker | ACEI/ARB vs. Control | β-blocker vs. Control |
| All-cause mortality | 580 (40.9) | 797 (33.8) | 723 (52.4) | 0.97 (0.87–1.09) | 0.78 (0.69–0.88)* | 0.81 (0.72–0.91)* |
| Aortic death | 125 (8.8) | 190 (8.1) | 129 (9.3) | 0.95 (0.75–1.21) | 0.99 (0.75–1.31) | 1.04 (0.80–1.35) |

Abbreviations: ACEI, angiotensin converting enzyme inhibitor; ARB, angiotensin receptor blocker; HR, hazard ratio; CI, confidence interval;

* *P* value <0.05.

© 2021 Chen SW et al. *JAMA Network Open*.

**eTable 6**. Baseline Characteristics of the Patients With Aortic Dissection According to the Use of ARBs or ACEIs

| | Data before IPTW‡ | | | | Data after IPTW† | | |
|---|---|---|---|---|---|---|---|
| Variable | ARB (*n* = 1,184) | ACEI (*n* = 480) | STD | | ARB (*n* = 1,184) | ACEI (*n* = 480) | STD |
| Age (years) | 68.5 ± 13.4 | 69.2 ± 13.7 | -0.05 | | 68.8 ± 13.5 | 68.8 ± 13.8 | 0.00 |
| Male gender | 787 (66.5) | 335 (69.8) | -0.07 | | 67.5% | 68.4% | -0.02 |
| Comorbid conditions | | | | | | | |
| Hypertension | | | | | | | |
| Non | 158 (13.3) | 88 (18.3) | -0.14 | | 14.7% | 14.0% | 0.02 |
| Without medication control | 312 (26.4) | 115 (24.0) | 0.06 | | 25.9% | 27.5% | -0.04 |
| With medication control | 714 (60.3) | 277 (57.7) | 0.05 | | 59.4% | 58.5% | 0.02 |
| Diabetes mellitus | 227 (19.2) | 84 (17.5) | 0.04 | | 18.4% | 18.4% | 0.00 |
| Congestive heart failure | 122 (10.3) | 72 (15.0) | -0.14 | | 11.2% | 10.7% | 0.02 |
| Coronary artery disease | 426 (36.0) | 191 (39.8) | -0.08 | | 36.9% | 37.8% | -0.02 |
| Old myocardial infarction | 70 (5.9) | 34 (7.1) | -0.05 | | 6.3% | 7.1% | -0.03 |
| Peripheral arterial disease | 81 (6.8) | 39 (8.1) | -0.05 | | 7.1% | 7.9% | -0.03 |
| Atrial fibrillation | 74 (6.3) | 26 (5.4) | 0.04 | | 6.0% | 5.6% | 0.02 |
| Old stroke | 211 (17.8) | 75 (15.6) | 0.06 | | 17.0% | 17.0% | 0.00 |
| Chronic kidney disease | 151 (12.8) | 67 (14.0) | -0.04 | | 12.6% | 11.3% | 0.04 |
| Dialysis | 12 (1.0) | 10 (2.1) | -0.09 | | 1.2% | 1.1% | 0.00 |
| COPD | 223 (18.8) | 97 (20.2) | -0.03 | | 19.3% | 19.4% | 0.00 |
| Asthma | 130 (11.0) | 47 (9.8) | 0.04 | | 10.7% | 10.5% | 0.01 |
| High degree AV blocker | 8 (0.7) | 3 (0.6) | 0.01 | | 0.7% | 0.5% | 0.02 |
| Charlson's total score | 2.0 [1.0, 3.0] | 2.0 [1.0, 4.0] | -0.06 | | 2.0 [1.0, 3.0] | 2.0 [1.0, 4.0] | -0.01 |
| Hospital level | | | | | | | |

© 2021 Chen SW et al. *JAMA Network Open*.

| | | | | | | | |
|---|---|---|---|---|---|---|---|
| Medical center (teaching hospital) | 720 (60.8) | 245 (51.0) | 0.20 | | 57.9% | 58.2% | -0.01 |
| Regional / district hospital | 464 (39.2) | 235 (49.0) | -0.20 | | 42.1% | 41.8% | 0.01 |
| Aortic surgery | | | | | | | |
| Non-surgery | 910 (76.9) | 397 (82.7) | -0.15 | | 78.7% | 80.3% | -0.04 |
| Type A | 218 (18.4) | 76 (15.8) | 0.07 | | 17.6% | 16.7% | 0.02 |
| Type B | 56 (4.7) | 7 (1.5) | 0.19 | | 3.8% | 2.9% | 0.05 |
| Extension of aortic surgery | | | | | | | |
| Ascending aorta replacement | 193 (16.3) | 63 (13.1) | 0.09 | | 15.4% | 14.6% | 0.02 |
| Aortic arch replacement | 54 (4.6) | 12 (2.5) | 0.11 | | 3.9% | 4.1% | -0.01 |
| Aortic root replacement | 5 (0.4) | 5 (1.0) | -0.07 | | 0.5% | 0.5% | 0.01 |
| Additional surgery | | | | | | | |
| CABG | 29 (2.4) | 16 (3.3) | -0.05 | | 2.6% | 2.5% | 0.01 |
| Valve replacement | 28 (2.4) | 16 (3.3) | -0.06 | | 2.6% | 2.5% | 0.01 |
| Valve repair | 98 (8.3) | 36 (7.5) | 0.03 | | 7.9% | 7.7% | 0.01 |
| Post OP anti-HTN medication | | | | | | | |
| CCB | 622 (52.5) | 253 (52.7) | 0.00 | | 52.5% | 52.7% | 0.00 |
| Alpha-blocker | 134 (11.3) | 67 (14.0) | -0.08 | | 12.0% | 11.8% | 0.01 |
| Thiazide | 75 (6.3) | 43 (9.0) | -0.10 | | 7.1% | 6.8% | 0.01 |
| Loop diuretics | 234 (19.8) | 118 (24.6) | -0.12 | | 20.8% | 19.6% | 0.03 |
| Potassium-sparing diuretics | 48 (4.1) | 16 (3.3) | 0.04 | | 3.7% | 3.3% | 0.02 |
| Vasodilator | 341 (28.8) | 165 (34.4) | -0.12 | | 30.1% | 31.9% | -0.04 |
| Nitrate | 244 (20.6) | 128 (26.7) | -0.14 | | 22.2% | 22.3% | 0.00 |
| Number of anti-HTN drugs (not including ACEI/ARB and β-blocker) | 1.4 ± 1.2 | 1.6 ± 1.2 | -0.18 | | 1.5 ± 1.2 | 1.5 ± 1.1 | 0.00 |

© 2021 Chen SW et al. *JAMA Network Open*.

| | | | | | | | |
|---|---|---|---|---|---|---|---|
| Post OP other medication | | | | | | | |
| Statin | 155 (13.1) | 32 (6.7) | 0.22 | | 11.3% | 11.5% | -0.01 |
| Antiplatelet | 379 (32.0) | 163 (34.0) | -0.04 | | 32.5% | 34.6% | -0.05 |
| Anticoagulant | 77 (6.5) | 45 (9.4) | -0.11 | | 7.1% | 7.1% | 0.00 |
| OHA | 139 (11.7) | 54 (11.3) | 0.02 | | 11.4% | 11.2% | 0.01 |
| Insulin | 19 (1.6) | 5 (1.0) | 0.05 | | 1.4% | 0.7% | 0.06 |
| Follow up year | 4.1 ± 3.1 | 5.3 ± 3.7 | -0.35 | | 4.6 ± 3.4 | 4.3 ± 3.4 | 0.08 |

Abbreviations: IPTW, inverse-probability-of-treatment weighting; ARB, angiotensin receptor blocker; ACEI, angiotensin converting enzyme inhibitor; STD, standardized difference; COPD, chronic obstructive pulmonary disease; AV, atrioventricular; CABG, coronary artery bypass graft; OP, operation; HTN, hypertension; CCB, calcium channel blocker; OHA, oral hypoglycemic agent;

‡ Value are given as number (%); † Values are given as %.

© 2021 Chen SW et al. *JAMA Network Open*.

**eTable 7.** Sensitivity Analysis by Treating Medication Use as Time-Varying Exposure (on Treatment) on the Primary Outcomes

| Outcome/ period | No. of event | Person-year | Incidence (95% CI)† | Adjusted HR (95% CI)* | *P* |
|---|---|---|---|---|---|
| **All-cause mortality** | | | | | |
| ACEI/ARB only without other anti-hypertensive agents | 126 | 6054.8 | 20.8 (17.2–24.4) | 0.91 (0.76–1.09) | 0.291 |
| BB only without other anti-hypertensive agents | 108 | 6947.5 | 15.6 (12.6–18.5) | 0.91 (0.74–1.12) | 0.366 |
| ACEI/ARB+BB without other anti-hypertensive agents | 106 | 10942.9 | 9.7 (7.8–11.5) | 0.68 (0.56–0.83) | <0.001 |
| Other anti-hypertensive medications | 2,451 | 119602.8 | 20.5 (19.7–21.3) | Reference | |
| **Aortic death** | | | | | |
| ACEI/ARB only without other anti-hypertensive agents | 45 | 6053.8 | 7.4 (5.3–9.6) | 0.84 (0.62–1.13) | 0.254 |
| BB only without other anti-hypertensive agents | 42 | 6946.7 | 6.1 (4.2–7.9) | 0.87 (0.63–1.19) | 0.382 |
| ACEI/ARB+BB without other anti-hypertensive agents | 42 | 10942.3 | 3.8 (2.7–5.0) | 0.64 (0.47–0.88) | 0.006 |
| Other anti-hypertensive medications | 981 | 119577.8 | 8.2 (7.7–8.7) | Reference | |

Abbreviations: ACEI, angiotensin converting enzyme inhibitor; ARB, angiotensin receptor blocker; BB, β-blocker; HR, hazard ratio; CI, confidence interval;

* All baseline characteristics, other anti-hypertensive agents and other medications listed in eTable 3 were adjusted;

† Number of event per 1,000 person-years.

© 2021 Chen SW et al. *JAMA Network Open*.

**eTable 8.** Sensitivity Analysis by Treating Medication Use as Time-Varying Exposure (on Treatment) on the Primary Outcomes, ARBs vs ACEIs

| Outcome/ period | No. of event | Person-year | Incidence (95% CI)† | Adjusted HR (95% CI) | *P* |
|---|---|---|---|---|---|
| **All-cause mortality** | | | | | |
| ACEI | 355 | 16507.6 | 21.5 (19.3–23.7) | Reference | Reference |
| ARB | 879 | 68067.0 | 12.9 (12.1–13.8) | 0.72 (0.63–0.82) | <0.001 |
| **Aortic death** | | | | | |
| ACEI | 154 | 16504.5 | 9.3 (7.9–10.8) | Reference | |
| ARB | 364 | 68059.1 | 5.4 (4.8–5.9) | 0.71 (0.58–0.87) | 0.001 |

Abbreviations: ACEI, angiotensin converting enzyme inhibitor; ARB, angiotensin receptor blocker; HR, hazard ratio; CI, confidence interval;

* All baseline characteristics, other anti-hypertensive agents and other medications listed in eTable 3 were adjusted;

† Number of event per 1,000 person-years.

© 2021 Chen SW et al. *JAMA Network Open*.

**eTable 9**. Baseline characteristics of the Patients With Aortic Dissection According to Antihypertensive Drug Use

| Variable | ACEI/ARB (*n* = 813) | β-blocker (*n* = 1,338) | CCB (*n* = 855) | *P* |
|---|---|---|---|---|
| Age (years) | 69.8 ± 13.9 | 62.8 ± 14.6 | 70.3 ± 13.2 | <0.001 |
| Male gender | 553 (68.0) | 918 (68.6) | 582 (68.1) | 0.947 |
| Comorbid conditions | | | | |
| Hypertension | | | | <0.001 |
| Non | 156 (19.2) | 415 (31.0) | 118 (13.8) | |
| Without medication control | 180 (22.1) | 409 (30.6) | 218 (25.5) | |
| With medication control | 477 (58.7) | 514 (38.4) | 519 (60.7) | |
| Diabetes mellitus | 151 (18.6) | 166 (12.4) | 151 (17.7) | <0.001 |
| Congestive heart failure | 117 (14.4) | 91 (6.8) | 79 (9.2) | <0.001 |
| Coronary artery disease | 341 (41.9) | 429 (32.1) | 267 (31.2) | <0.001 |
| Old myocardial infarction | 58 (7.1) | 57 (4.3) | 40 (4.7) | 0.011 |
| Peripheral arterial disease | 58 (7.1) | 76 (5.7) | 45 (5.3) | 0.231 |
| Atrial fibrillation | 69 (8.5) | 79 (5.9) | 54 (6.3) | 0.058 |
| Old stroke | 137 (16.9) | 134 (10.0) | 157 (18.4) | <0.001 |
| Chronic kidney disease | 111 (13.7) | 180 (13.5) | 153 (17.9) | 0.010 |
| Dialysis | 5 (0.6) | 16 (1.2) | 20 (2.3) | 0.008 |
| COPD | 194 (23.9) | 124 (9.3) | 148 (17.3) | <0.001 |
| Asthma | 93 (11.4) | 69 (5.2) | 74 (8.7) | <0.001 |
| High degree AV blocker | 7 (0.9) | 5 (0.4) | 3 (0.4) | 0.229 |
| Charlson's total score | 2.0 [2.0, 4.0] | 2.0 [1.0, 3.0] | 2.0 [1.0, 4.0] | <0.001 |
| Hospital level | | | | <0.001 |

© 2021 Chen SW et al. *JAMA Network Open*.

| | | | | |
|---|---|---|---|---|
| Medical center (teaching hospital) | 431 (53.0) | 869 (64.9) | 457 (53.5) | |
| Regional / district hospital | 382 (47.0) | 469 (35.1) | 398 (46.5) | |
| Aortic surgery | | | | <0.001 |
| Non-surgery | 637 (78.4) | 775 (57.9) | 635 (74.3) | |
| Type A | 149 (18.3) | 498 (37.2) | 182 (21.3) | |
| Type B | 27 (3.3) | 65 (4.9) | 38 (4.4) | |
| Extension of aortic surgery | | | | |
| Ascending aorta replacement | 130 (16.0) | 402 (30.0) | 147 (17.2) | <0.001 |
| Aortic arch replacement | 37 (4.6) | 125 (9.3) | 49 (5.7) | <0.001 |
| Aortic root replacement | 7 (0.9) | 52 (3.9) | 7 (0.8) | <0.001 |
| Additional surgery | | | | |
| CABG | 30 (3.7) | 113 (8.4) | 32 (3.7) | <0.001 |
| Valve replacement | 32 (3.9) | 108 (8.1) | 18 (2.1) | <0.001 |
| Valve repair | 60 (7.4) | 236 (17.6) | 89 (10.4) | <0.001 |
| Post OP anti-HTN medication | | | | |
| Alpha-blocker | 68 (8.4) | 89 (6.7) | 95 (11.1) | 0.001 |
| Thiazide | 49 (6.0) | 57 (4.3) | 27 (3.2) | 0.016 |
| Loop diuretics | 199 (24.5) | 344 (25.7) | 164 (19.2) | 0.002 |
| Spironolactone (Potassium-sparing ) | 38 (4.7) | 39 (2.9) | 31 (3.6) | 0.104 |
| Vasodilator | 277 (34.1) | 326 (24.4) | 212 (24.8) | <0.001 |
| Nitrate | 205 (25.2) | 246 (18.4) | 147 (17.2) | <0.001 |
| Number of anti-HTN drugs | 2.0 ± 1.1 | 1.8 ± 1.0 | 1.8 ± 1.0 | <0.001 |
| Post OP other medication | | | | |
| Statin | 96 (11.8) | 118 (8.8) | 70 (8.2) | 0.024 |

© 2021 Chen SW et al. *JAMA Network Open*.

| | | | | |
|---|---|---|---|---|
| Antiplatelet (aspirin or clopidogrel) | 309 (38.0) | 357 (26.7) | 214 (25.0) | <0.001 |
| Anticoagulant | 90 (11.1) | 215 (16.1) | 52 (6.1) | <0.001 |
| OHA | 92 (11.3) | 90 (6.7) | 86 (10.1) | 0.001 |
| Insulin | 9 (1.1) | 8 (0.6) | 7 (0.8) | 0.436 |
| Follow up year | 4.4 ± 3.4 | 4.7 ± 3.4 | 4.1 ± 3.3 | <0.001 |

Abbreviations: ACEI, angiotensin converting enzyme inhibitor; ARB, angiotensin receptor blocker; COPD, chronic obstructive pulmonary disease; AV, atrioventricular; CABG, coronary artery bypass graft; OP, operation; HTN, hypertension; CCB, calcium channel blocker; OHA, oral hypoglycemic agent;

Value are given as number (%), median [25$^{th}$, 75$^{th}$ percentile] or mean ± standard deviation.

© 2021 Chen SW et al. *JAMA Network Open*.

**eTable 10.** Outcomes of Primary Interest During the Follow-up Comparing ACEIs/ARBs, β-Blockers and CCBs Users

| | Number of event (%) | | | | Propensity scores adjusted HR (95% CI) | | | | |
|---|---|---|---|---|---|---|---|---|---|
| Outcome | ACEI/ARB (*n* = 813) | β-blocker (*n* = 1,338) | CCB (*n* = 855) | | ACEI/ARB vs. β-blocker | | ACEI/ARB vs. CCB | | β-blocker vs. CCB |
| All-cause mortality | 317 (39.0) | 422 (31.5) | 380 (44.4) | | 0.88 (0.75–1.02) | | 0.76 (0.65–0.88)* | | 0.86 (0.75–0.995)* |
| Aortic death | 59 (7.3) | 106 (7.9) | 71 (8.3) | | 0.67 (0.48–0.94)* | | 0.70 (0.49–1.01) | | 1.05 (0.77–1.43) |

Abbreviations: ACEI, angiotensin converting enzyme inhibitor; ARB, angiotensin receptor blocker; CCB, calcium channel blocker; HR, hazard ratio; CI, confidence interval;

* *P* value <0.05.

© 2021 Chen SW et al. *JAMA Network Open*.

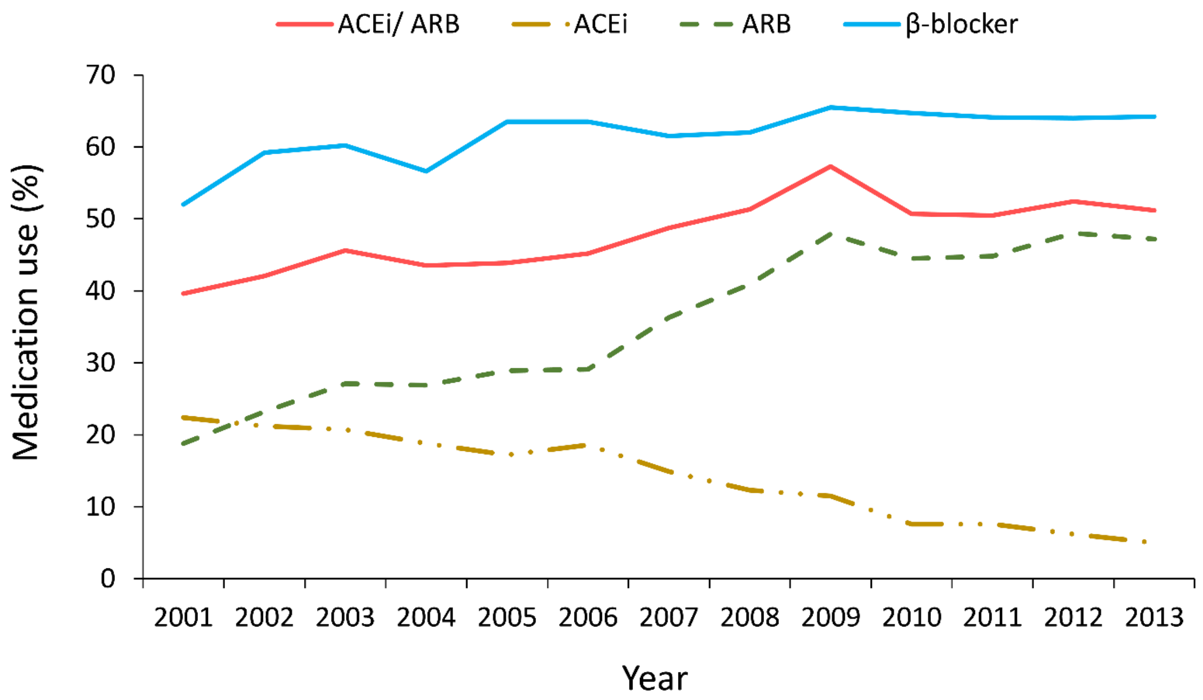


**eFigure 1.** The Trend of Prescription for Antihypertensive Agents of Primary Interest Across 2001 to 2013 in Taiwan

© 2021 Chen SW et al. *JAMA Network Open*.

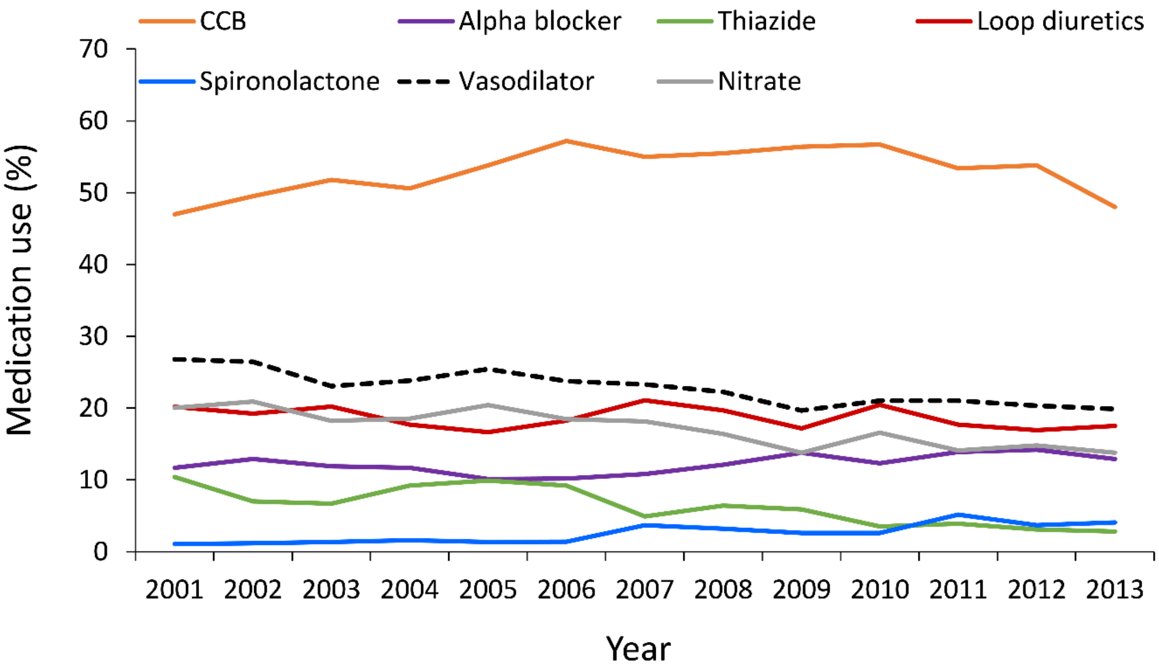


**eFigure 2.** The Trend of Prescription for Antihypertensive Agents Not of Primary Interest Across 2001 to 2013 in Taiwan

© 2021 Chen SW et al. *JAMA Network Open*.

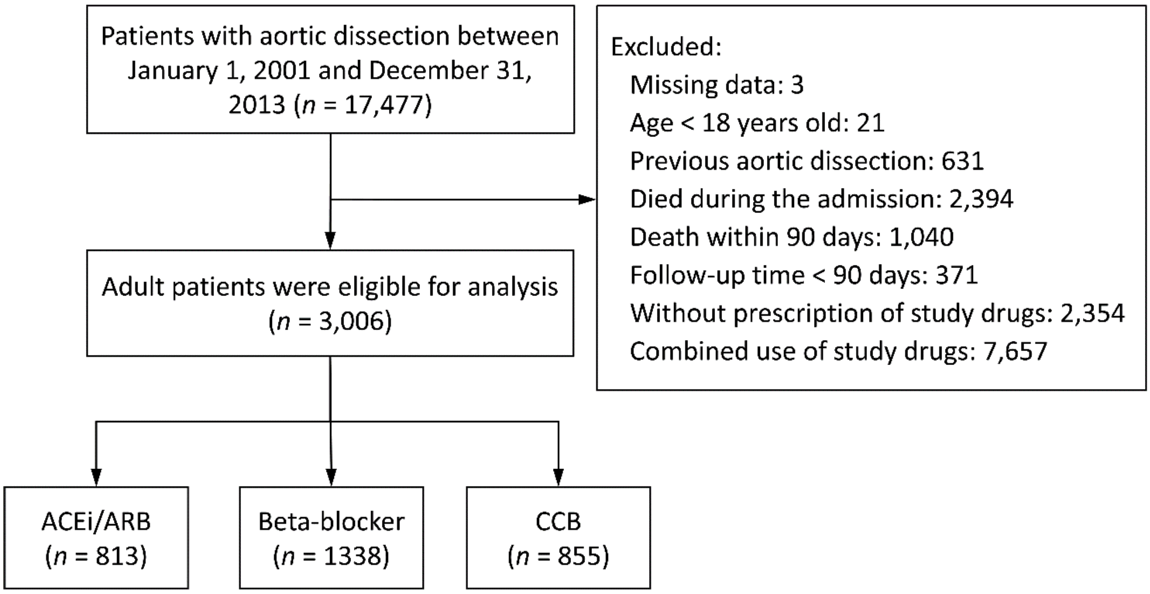


**eFigure 3.** The Flowchart for Inclusion of Patients With Use of ACEIs/ARBs, β-Blockers or CCBs Arm

© 2021 Chen SW et al. *JAMA Network Open*.
